# Clinical practice guidelines and consensus statements integrating periodontal disease into cardiology, diabetes care and dementia: A scoping review and gap analysis

**DOI:** 10.4317/medoral.27981

**Published:** 2026-01-24

**Authors:** Fernando Mauricio Espada-Salgado, Stefan Vasile Stefanescu, María Mihaela Iuga

**Affiliations:** 1 School of dentistry, Faculty of Health Sciences, Private University of Tacna, Tacna, Peru; 2 Top Smiles, Liverpool L6 4DU, Merseyside, UK

## Abstract

**Background:**

Periodontal disease (PD) is a prevalent chronic inflammatory condition linked to cardiovascular disease, diabetes mellitus and dementia, yet it is unclear how far medical specialty guidelines translate this evidence into concrete recommendations for assessment, referral and shared care. Objective: To map clinical practice guidelines, consensus statements and position papers that contain explicit, actionable recommendations on PD within major medical specialties.

**Material and Methods:**

A scoping review was conducted following Joanna Briggs Institute guidance and reported according to PRISMA-ScR. PubMed/MEDLINE, Scopus, Web of Science Core Collection and LILACS were searched from 2005 to 2025 using MeSH/DeCS and free-text terms for periodontal disease, guideline documents and target specialties. Records were de-duplicated, screened in Rayyan by two independent reviewers and included if they were peer-reviewed or professionally endorsed guidance for adults that contained explicit periodontal recommendations. Data were charted in a standardized matrix and synthesized descriptively by specialty.

**Results:**

The search yielded a small corpus of eligible documents. Operational recommendations were concentrated in diabetes-periodontitis statements, with fewer detailed pathways in cardiovascular and dementia care and very limited or absent guidance in obstetrics/gynecology and rheumatology. Across documents, PD was framed as a modifiable risk or complication, but periodontal definitions, screening intervals and division of responsibilities between medical and dental providers were heterogeneous or poorly specified.

**Conclusions:**

Few medical specialty guidelines formally integrate PD into chronic disease care. Harmonized, multidisciplinary guidance with standardized definitions, clear referral criteria and shared follow-up schedules is needed to embed periodontal health within noncommunicable disease management.

## Introduction

Periodontal disease (PD) is a chronic inflammatory condition affecting the tooth-supporting tissues and is among the most prevalent non-communicable diseases worldwide (1 , 2). Severe forms affect approximately 10-19% of the global population aged 15 years and represent a major cause of tooth loss, particularly in individuals with cardiovascular disease, diabetes mellitus, smoking habits and socioeconomic disadvantage (3). This substantial burden has positioned PD as a relevant public health problem with implications beyond oral health.

Increasing evidence indicates that PD has systemic consequences. Periodontal dysbiosis and chronic inflammation promote transient bacteremia and persistent low-grade systemic inflammatory responses, which may contribute to endothelial dysfunction and atherogenesis (4 , 5). Meta-analyses and umbrella reviews consistently report an association between PD and cardiovascular disease, including coronary heart disease and stroke, supporting the concept of PD as a potentially modifiable cardiovascular risk factor (6).

PD is also closely linked to diabetes mellitus through a bidirectional relationship, whereby hyperglycemia increases susceptibility to periodontal tissue destruction and periodontal inflammation adversely affects glycemic control; interventional evidence suggests that periodontal therapy can lead to modest but clinically relevant improvements in glycated hemoglobin (HbA1c) (7).

Beyond cardiometabolic conditions, PD and tooth loss have been associated with cognitive impairment and dementia. Meta-analyses and longitudinal studies suggest an increased risk of cognitive decline among individuals with periodontitis, although the certainty of evidence remains limited and causality cannot be definitively established (8 , 9). Proposed mechanisms include chronic systemic inflammation, vascular dysfunction, dissemination of periodontal pathogens and the functional consequences of impaired mastication (10).

Despite this accumulating evidence linking PD with cardiovascular disease, diabetes and cognitive disorders, it remains unclear to what extent these associations have been translated into explicit and operational recommendations within medical specialty guidelines. This scoping review therefore aims to map clinical practice guidelines, consensus statements and position papers that integrate PD into cardiology, diabetes care and dementia management, and to identify areas in which formal guidance is lacking.

## Material and Methods

Methodological approach

This scoping review followed Joanna Briggs Institute (JBI) methodology, which is appropriate for mapping broad and heterogeneous evidence and for identifying gaps to inform practice, policy and research (11). Reporting adhered to the PRISMA-ScR checklist and explanation (12). In line with JBI guidance, no formal risk-of-bias appraisal or meta-analysis was undertaken; findings are presented descriptively.

Protocol and registration

The protocol was prospectively registered on the Open Science Framework (OSF) before screening. The registration record, full search strategies, screening logs, data-charting templates and analytic outputs are (or will be) available in the OSF project (registration DOI: https://osf.io/jfqsg). Any deviations from the protocol will be documented but do not alter the objectives or eligibility criteria.

Review question and PCC framework

The review was structured using the Population-Concept-Context (PCC) framework recommended by JBI (11).

Population: Adults (18 years) with periodontal disease (gingivitis or periodontitis) or at risk of periodontal disease who are being managed in medical specialties.

Concept: Clinical practice guidelines, consensus statements, position statements or other structured guidance that provide explicit, actionable recommendations on periodontal disease (e.g. screening, risk communication, referral, treatment timing, co-management).

Context: Medical care in which periodontal disease is integrated into systemic disease management, with a primary focus on cardiology, diabetes care/endocrinology and dementia/geriatric neurology. Obstetrics/gynecology and rheumatology were also searched, but no eligible documents were identified.

Review question

Which clinical practice guidelines, consensus statements and related guidance documents published from January 2005 to December 2025 integrate periodontal disease into cardiology, diabetes care and dementia management, and how do they operationalize screening, referral and shared care between dental and medical teams?

Eligibility criteria

Inclusion criteria

We included peer-reviewed documents that:

Were clinical practice guidelines, consensus or position statements, practice parameters, policy statements or similar structured guidance formally developed or endorsed by professional societies, scientific organizations or multidisciplinary expert panels.

Addressed adults with periodontal disease or adults with systemic conditions in whom periodontal disease was discussed with explicit recommendations.

Provided actionable clinical guidance on periodontal disease for medical and/or dental professionals (e.g. risk assessment, screening, education, referral, treatment timing, follow-up, co-management).

Were framed within cardiology/cardiovascular medicine, diabetes care/endocrinology or dementia/geriatric neurology in any healthcare setting; guidance from other specialties was eligible if all other criteria were met.

Were published between 1 January 2005 and 1 December 2025 in English or with an English version available.

Exclusion criteria

We excluded: Primary research studies (trials, observational studies, prediction models), case reports, narrative reviews, systematic reviews, meta-analyses, editorials or commentaries without structured recommendations; documents that mentioned periodontal disease only as background without explicit guidance; pediatric-only or school-based programs without extractable adult recommendations; guidance focused only on general oral health or caries; self-labelled "guidelines" lacking professional endorsement or a formal development/consensus process; conference abstracts, posters or grey literature without peer-reviewed full text; duplicate or superseded versions (keeping the most complete and recent); and records for which full text could not be obtained.

Information sources and search strategy

Four databases were searched systematically: Scopus, Web of Science Core Collection, PubMed/MEDLINE and LILACS within the Virtual Health Library (BVS). Searches combined controlled vocabulary (MeSH, DeCS) and free-text terms tailored to each source. Example PubMed syntax:

("periodontal disease" OR "periodontitis" OR "gingivitis") AND ("guideline" OR "practice guideline" OR "consensus statement" OR "position statement" OR "policy statement" OR "practice parameter") AND (cardiology OR neurology OR dementia OR endocrinology OR diabetes OR obstetrics OR gynecology OR rheumatology).

NOT terms and year limits (2005-2025) were applied to exclude systematic reviews, meta-analyses and umbrella reviews. Searches were last updated on 1 December 2025. Reference lists of included documents and relevant reviews were screened for additional eligible guidelines. Full search strings and any refinements are available in the OSF project.

Selection of sources of evidence

All records were exported to reference management software for de-duplication and imported into Rayyan for screening. Across databases, 128 records were retrieved (Scopus n=47, Web of Science n=14, PubMed n=44, LILACS n=23); after removing duplicates, 87 unique records remained.

Two reviewers independently screened titles and abstracts against the eligibility criteria in Rayyan; any record considered potentially relevant by either reviewer proceeded to full-text assessment. The same reviewers then assessed full texts independently; disagreements were resolved by discussion and, when needed, a third reviewer. Reasons for full-text exclusion were recorded. Fourteen documents were assessed in full text, of which six met all inclusion criteria and eight were excluded (e.g. narrative reviews, non-operational policy statements, pediatric-only documents). The selection process will be summarized in a PRISMA-ScR flow diagram.

Data charting

A piloted data-charting form was used to extract, for each included document: Bibliographic details; issuing body; document type; medical specialty and target population; definition and description of periodontal disease; scope and objectives; periodontal-related recommendations (screening and risk assessment, timing and setting of dental evaluation, referral pathways, treatment criteria, maintenance, patient education, interprofessional collaboration); methods of guideline development; and any implementation tools or statements on resource use.

One reviewer charted all data and a second independently verified each record; discrepancies were resolved by consensus or third-reviewer arbitration. A per-record matrix summarized citation details, issuing body, specialty, development methodology and the presence or absence of key periodontal recommendations (screening, referral, treatment timing, maintenance, education, interprofessional coordination). This matrix will be provided as a supplementary table.

Synthesis of results

Findings were synthesized narratively and in summary tables, grouped primarily by medical specialty (cardiology, diabetes care, dementia). Within each specialty, we compared how guidelines conceptualized the PD-systemic relationship, which tasks were assigned to medical versus dental professionals, and how screening, referral and co-management were operationalized, as well as temporal and geographical patterns. Consistent with JBI recommendations for scoping reviews (11), no formal critical appraisal, effect-size estimation or quantitative synthesis was undertaken; the focus was on describing the breadth and content of existing guidance and identifying evidence and policy gaps, particularly in specialties where no eligible guidelines were found.

Data availability

All materials supporting this review including complete search strategies, screening logs, data-charting templates, the PRISMA-ScR checklist and analytic outputs are (or will be) openly available in the OSF project for this review (registration DOI: https://osf.io/jfqsg).

## Results

Study selection

**Figure 1:**
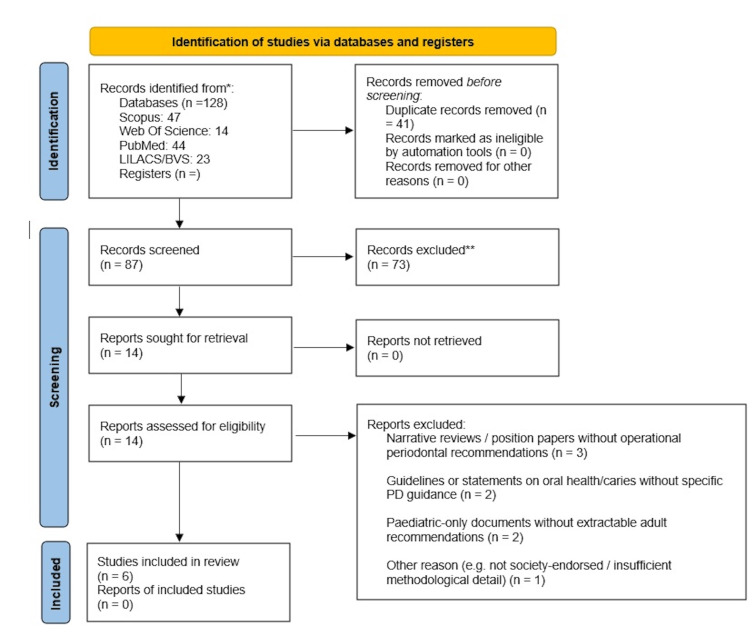
PRISMA 2020 flow diagram for the scoping review. Searches conducted on 1 December 2025.

The search retrieved 128 records (Scopus=47, Web of Science=14, PubMed=44, LILACS/BVS=23). After removing 41 duplicates, 87 unique records were screened and 73 were excluded at title/abstract level. Fourteen fulltext documents were assessed for eligibility; eight were excluded because they were narrative reviews or position papers without operational periodontal guidance, pediatric documents or lacked endorsement by professional societies. Six documents met the inclusion criteria and were retained for data charting. The PRISMA flow diagram is shown in Figure 1.

1

Characteristics of included guidance

Table 1 summarizes the six included documents.

Table 1

Three were consensus guidelines linking periodontitis with diabetes care, two addressed cardiovascular medicine (one editors' consensus and one perioperative consensus), and one provided a guideline for oral health care in people with dementia. No eligible guidance was identified in obstetrics/gynecology or rheumatology. The documents were issued between 2006 and 2021 and originated from international or national professional societies. All provided explicit recommendations relevant to screening/risk assessment, referral, and medical-dental co-management.

Descriptive mapping of periodontal-related recommendations

Across the included guidance, periodontal content was addressed through a common set of actionable domains. Domains identified included: Risk communication (informing patients about periodontal-systemic links); periodontal assessment or oral-risk assessment within the clinical pathway; referral and coordination mechanisms between medical and dental professionals; timing considerations for dental/periodontal management (particularly in peri-procedural cardiovascular settings); follow-up orientation including periodontal review or maintenance planning and patient/caregiver education and support (notably in dementia care).

The level of operational detail reported differed by document type and medical area. In the three diabetes-related documents, structured elements were commonly reported, including routine periodontal assessment, bidirectional referral, and maintenance orientation within chronic disease management. The two cardiovascular documents mainly reported recommendations on coordination and pre-procedural dental/periodontal screening to manage oral infection foci. The dementia guideline emphasized oral-risk assessment and caregiver-supported oral hygiene and care planning.

## Discussion

This scoping review identified only six formal guidance documents integrating periodontal disease (PD) into cardiology, diabetes care or dementia pathways, underscoring a translational gap between a substantial global disease burden and the limited availability of operational, specialty-facing recommendations (1 , 2). This gap is particularly relevant given the consistent evidence base supporting links between PD and cardiovascular disease, diabetes and cognitive impairment (3 - 10). With Results presented as a descriptive mapping of the eligible guidance, this discussion provides the interpretive synthesis by specialty, highlighting convergences, differences and implementation implications across pathways.

Cardiology

Cardiovascular guidance comprised two consensus-based documents: An editors' consensus framing periodontitis as a marker of vascular risk and emphasizing risk communication and interprofessional collaboration, and a peri-operative RAND/UCLA consensus recommending systematic dental assessment and management of oral infection foci before cardiothoracic and interventional cardiovascular procedures with coordinated timing between dental and cardiac teams; overall, PD is operationalized mainly through referral/coordination mechanisms rather than standardized periodontal metrics (14 , 15). However, operational specificity for routine implementation remains limited, with inconsistent periodontal definitions, staging and actionable referral thresholds. Given contemporary evidence describing oral health as a modifiable cardiovascular risk factor and umbrella-level synthesis supporting PD-CVD associations, future specialty-facing guidance would benefit from clearer minimum datasets for periodontal assessment, explicit referral triggers and delineation of responsibilities between medical and dental teams (4 - 6).

Diabetes

Guidance at the diabetes-periodontitis interface was comparatively more mature, with international and national consensus documents recommending routine periodontal assessment, structured referral pathways and coordinated medical-dental care; these documents converge on implementable endpoints including systematic identification of periodontal inflammation in people with diabetes, bidirectional referral and planned maintenance within chronic disease management (16 - 18). This aligns with the established bidirectional relationship between periodontitis and diabetes and the rationale that periodontal therapy may contribute to modest improvements in glycemic control (7). Despite this maturity, implementation gaps persist, including heterogeneity in referral thresholds and follow-up schedules and limited embedding of periodontal indicators into diabetes workflows or quality metrics; priorities for translation include standardizing referral criteria, minimum follow-up schedules and shared-care responsibilities without adding diagnostic burden (16 - 18).

Dementia and geriatrics

Dementia and geriatric care were addressed by only one eligible guideline, which emphasized oral-risk assessment, caregiver training and stage-adapted care planning to preserve comfort, function and dignity (13). This scarcity is notable given evidence linking PD and tooth loss with cognitive decline and dementia, including systematic review and meta-analytic findings and recent narrative synthesis (3 , 8 - 10). The available guidance remains predominantly dental-led and oriented toward care delivery rather than embedded within medical dementia pathways, highlighting an opportunity to translate current evidence into operational checkpoints (e.g., triggers for dental referral) and feasible follow-up schedules within long-term care and geriatric services.

Evidence-guidance gaps beyond the mapped pathways

Beyond cardiology, diabetes and dementia, evidence continues to document broader systemic relevance of PD, including reports addressing PD in systemic genetic disorders (19) and systematic review evidence linking PD with adverse pregnancy outcomes (20). Broader overviews of periodontitis and systemic diseases further emphasize shared inflammatory pathways and interprofessional management needs, while epidemiological work highlights PD prevalence, comorbid associations and prevention implications at a population level (21 - 23). In the cognitive domain, recent narrative synthesis has discussed links between periodontitis and Alzheimer's disease (24). Regarding obstetrics, additional reviews summarize two decades of research and overviews of systematic reviews on PD and pregnancy outcomes, noting that associations are frequently reported but residual confounding and variability in effect estimates remain important considerations (25 , 26). Forward-looking perspectives also emphasize the need for higher-quality studies and more pragmatic clinical translation in this area (27). Nevertheless, society-endorsed, risk-stratified and operational guidance in obstetrics/gynecology, rheumatology and other specialties remains largely absent, reinforcing that the principal deficit is not the lack of association studies but rather limited translation into implementable, specialty-facing recommendations.

Practical implications

To facilitate implementation and to provide a unified practical summary, Table 2 offers a one-page "quick reference" across specialties, summarizing actionable endpoints, how periodontal disease is operationalized, and pragmatic "do/refer/follow-up" elements extracted from the included guidance documents. This table is a descriptive synthesis and does not constitute a new clinical guideline or any formal grading of recommendations.

Table 2

Strengths, limitations and implications for future guidance

This review followed JBI methodological guidance and PRISMA-ScR reporting standards, providing a structured map of formal guidance rather than effect-size synthesis (11 , 12). A limitation inherent to scoping reviews is that recommendations are summarized descriptively without formal certainty appraisal; however, this approach is appropriate for identifying where operational guidance exists and where it is absent. Future multidisciplinary documents should adopt standardized periodontal definitions, specify referral thresholds and follow-up schedules, and clearly delineate responsibilities across medical and dental teams to enable implementation. Strengthening periodontal-systemic disease education in training programs may facilitate interprofessional care and improve uptake of integrated pathways (28).

## Conclusions

This scoping review indicates that, despite consistent evidence of associations between periodontal disease and cardiovascular disease, diabetes and cognitive impairment, very few medical guidelines explicitly integrate periodontal assessment and management into routine care pathways. The most developed and operational recommendations are found in joint diabetes-periodontology statements, whereas cardiovascular and dementia guidance remains comparatively less detailed and less standardized. No eligible society-endorsed guidance was identified for obstetrics/gynecology or rheumatology, highlighting important gaps in the integration of periodontal health within systemic disease management.

These findings support the need for closer collaboration between medical and dental organizations when developing clinical practice guidance. Future documents should align on standard periodontal classifications, define clear roles for screening and referral, and provide practical implementation tools (e.g., referral triggers, minimum datasets and follow-up schedules) adaptable to diverse health-system settings. Strengthening periodontal integration within chronic-disease care pathways may help improve coordinated care, preserve function and quality of life, and reduce the overall burden of non-communicable disease.

## Figures and Tables

**Table 1:** Table Summary of medical specialty guidelines including periodontal-related recommendations.

No.	First author / year - short title	Issuing body / country	Medical area	Main periodontal -related recommendations
1	Fiske et al., 2006 - Oral health care in dementia [13]	British Society for Disability and Oral Health, UK	Dementia/geriatrics	Equal right to oral health; train carers and dental teams; use oral-risk assessment; plan and adapt periodontal care according to dementia stage.
2	Friedewald et al., 2009 - AJC &J Periodontol editors' consensus [14]	Editors of American Journal of Cardiology and Journal of Periodontology, USA	Cardiology	Inform patients with moderate-severe periodontitis about increased CVD risk; recommend medical evaluation when CVD risk factors are present; encourage collaboration between physicians and periodontists.
3	Cotti et al., 2019 - Perioperative dental screening [15]	Six Italian scientific societies (cardiology, surgery, periodontology, endodontics)	Cardiology/perioperative	Standardized dental/periodontal screening before cardiothoracic or interventional procedures; decision tables linking oral infections to treatments and timing; emphasizes dentist-cardiologist coordination.
4	Sanz et al., 2018 - IDF/EFP diabetes-periodontitis guidelines [16]	International Diabetes Federation & European Federation of Periodontology	Diabetes/systemic medicine	Physicians to educate diabetic patients on periodontal risk, ask about diagnosis/symptoms and refer; recommend annual periodontal review; dentists to assess glycemic control, give tailored oral-hygiene advice and coordinate with physicians.
5	Jain et al., 2020 - Periodontal disease in diabetes (GCP) [17]	Indian Society of Periodontology & Research Society for the Study of Diabetes in India	Diabetes/endocrinology	Dentists to probe and stage periodontitis; recognize 'red flags'; screen for prediabetes; refer to physicians; aim for gingival health and >=20 teeth with regular maintenance.
6	Adda et al., 2021 - SID-SIdP-AMD joint consensus [18]	Italian diabetology & periodontology societies	Diabetes/endocrinology	Diabetologists to warn about periodontal risk, ask about signs and refer; advise annual dental check-ups; dentists to educate on systemic links, record medical history/BMI, suggest diabetes screening in severe periodontitis and manage therapy-related risks.

1

**Table 2:** Table Quick reference of periodontal endpoints and operational actions across specialties.

Pathway	Periodontal endpoint(s) addressed	What is operationalized (actionable elements)	Practical 'do / refer / follow-up' summary
Cardiology	PD as risk marker; oral infection foci before cardiac procedures	Risk communication + interprofessional coordination; pre-procedural dental/periodontal screening and infection focus management	Do: inform patients with periodontitis about potential higher CVD risk and encourage coordination of care. Refer/plan: dental/periodontal assessment when PD is present and especially before cardiothoracic/interventional procedures; manage oral infection foci and coordinate timing with cardiac team. Gap: limited standardized PD definitions/staging and referral thresholds for routine cardiology workflows. [14,15]
Diabetes	PD as comorbidity/complication; bidirectional risk; co-management	Routine periodontal assessment; structured referral pathways; coordinated medical-dental care; maintenance orientation	Do: incorporate periodontal evaluation into diabetes care and educate on bidirectional link. Refer/plan: systematic dental/periodontal referral (at diagnosis and follow-up), plus coordinated management between physicians and dentists. Follow-up: planned periodontal maintenance (often operationalized as regular/annual review depending on risk). Gap: heterogeneous thresholds and follow-up schedules; periodontal indicators not consistently embedded into diabetes care metrics/workflows. [16-18]
Dementia / geriatrics	Oral-risk assessment; caregiver-supported oral hygiene; stage-adapted care planning	Caregiver training; risk-based planning and adaptation by dementia stage; focus on comfort/function and access to dental care	Do: implement oral-risk assessment and caregiver training to maintain oral hygiene. Plan: stage-adapted preventive/maintenance care and facilitate dental access for care-dependent patients. Gap: only one formal guidance identified; limited integration into medical dementia pathways and lack of standardized triggers/schedules. [13]

This table summarizes explicit operational recommendations reported in the included guidance documents; it does not constitute new guidance or recommendation grading. PD: Periodontal disease; CVD: Cardiovascular disease.

## Data Availability

Declared none.
